# Genome-Wide Approaches to *Drosophila* Heart Development

**DOI:** 10.3390/jcdd3020020

**Published:** 2016-05-27

**Authors:** Manfred Frasch

**Affiliations:** Division of Developmental Biology, Department of Biology, Friedrich-Alexander University of Erlangen-Nürnberg, Staudtstr. 5, Erlangen 91058, Germany; manfred.frasch@fau.de; Tel.: +49-9131-8528061

**Keywords:** cardiogenesis, heart development, cardiogenic transcription factors, genetic screens, genomics, transcriptomics, ChIP-chip, ChIP-seq, machine learning

## Abstract

The development of the dorsal vessel in *Drosophila* is one of the first systems in which key mechanisms regulating cardiogenesis have been defined in great detail at the genetic and molecular level. Due to evolutionary conservation, these findings have also provided major inputs into studies of cardiogenesis in vertebrates. Many of the major components that control *Drosophila* cardiogenesis were discovered based on candidate gene approaches and their functions were defined by employing the outstanding genetic tools and molecular techniques available in this system. More recently, approaches have been taken that aim to interrogate the entire genome in order to identify novel components and describe genomic features that are pertinent to the regulation of heart development. Apart from classical forward genetic screens, the availability of the thoroughly annotated *Drosophila* genome sequence made new genome-wide approaches possible, which include the generation of massive numbers of RNA interference (RNAi) reagents that were used in forward genetic screens, as well as studies of the transcriptomes and proteomes of the developing heart under normal and experimentally manipulated conditions. Moreover, genome-wide chromatin immunoprecipitation experiments have been performed with the aim to define the full set of genomic binding sites of the major cardiogenic transcription factors, their relevant target genes, and a more complete picture of the regulatory network that drives cardiogenesis. This review will give an overview on these genome-wide approaches to *Drosophila* heart development and on computational analyses of the obtained information that ultimately aim to provide a description of this process at the systems level.

## 1. Introduction

**Figure 1:**
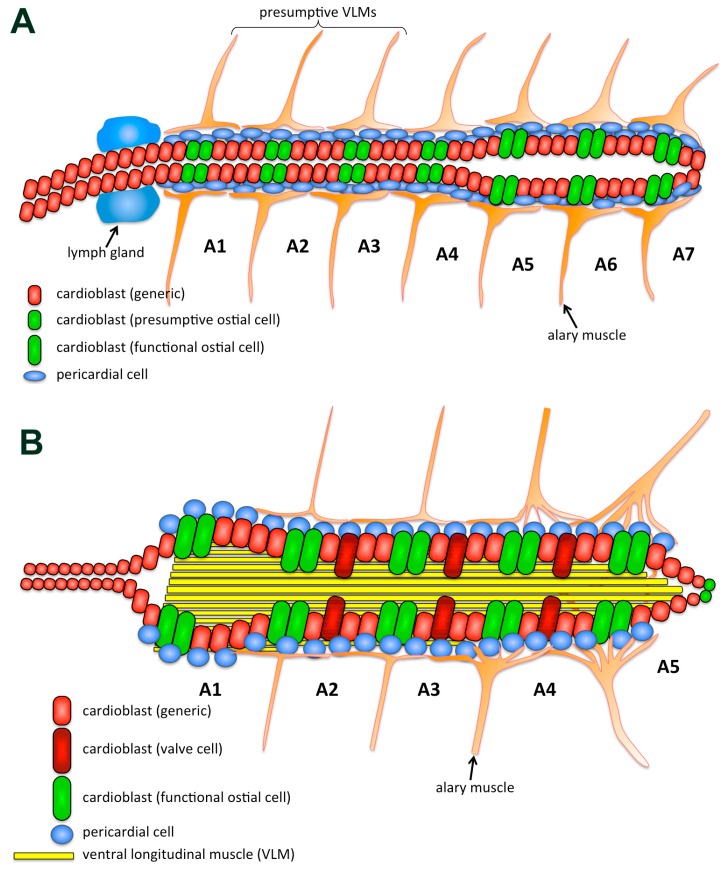
Schematic drawings of dorsal vessels from late stage *Drosophila* embryo (**A**) and adult fly (**B**) (not to scale). The cell types discussed in the text are color-coded as indicated. The adult heart is remodeled from the larval dorsal vessel, which involves histolysis of cells from abdominal segments A6 and A7, transdifferentiation of the three anterior pairs of alary muscles into ventral longitudinal muscles (VLMs), differentiation of presumptive ostial cells from the larval aorta into functional ostia, formation of three valves, formation of the conical heart chamber, and a reduction in the number of pericardial cells.

Studies on the specification and early development of the *Drosophila* heart (more accurately known as dorsal vessel; Figure 1) have provided one of the first examples for the regulatory circuits guiding cardiogenesis. The insights from *Drosophila* have also produced key inputs into studies on the molecular control of vertebrate heart development and resulted in important advances in this field. The findings from these studies provided a basic framework of the intersecting signaling and transcriptional networks and their temporal and spatial integration that control early heart development. Similar approaches have also shed light on later processes of heart morphogenesis and differentiation [1]. Although these studies in *Drosophila* have been highly successful, they have relied heavily on candidate approaches and fortuitous discoveries, often combined with reverse genetics, which led to the identification of signaling processes and of new members of transcription factor families that play key roles during *Drosophila* cardiogenesis. However, it is evident that without more systematic approaches, many important regulatory genes and processes will be missed, thus leading to an incomplete picture of the regulation of heart development. Due to the availability of highly developed and easily implemented genetic techniques, *Drosophila* is in fact predestined for systematic and unbiased genetic screens that interrogate the entire genome. Apart from classical chemical or insertional mutagenesis screens, the availability of the fully sequenced genome of *Drosophila* since the year 2000 [2] and its thorough annotation has opened additional avenues for genome-wide approaches. These include functional screens via systematic RNA interference (RNAi). Importantly, they made genomic approaches possible that allow genome-wide searches for novel regulators and provide descriptions of global events of gene regulation during cardiogenesis. These comprise analyses of the transcriptomes and proteomes of the developing heart at different stages and under different conditions, as well as genome-wide screens for the binding sites of cardiogenic transcription factors that had been described in earlier studies. Increasingly sophisticated computational tools have been instrumental in teasing out a wealth of interesting information from these datasets. All this information can now be employed in follow-up investigations and integrated into the existing framework, which will lead to a much more complete picture of the events that control heart development in *Drosophila*.

## 2. Genetic Screens for Mutants Affecting Heart Development

Forward genetic screens for mutations affecting a particular developmental pathway provide an unbiased approach to identify novel components with critical functions in this process. The power of such screens has been highlighted by the extraordinary success of screens for ethyl methanesulfonate (EMS)-induced mutations that affect axis formation and segmentation in the early *Drosophila* embryo [3]. Analogous screens have been performed for EMS-induced mutations that affect the *Drosophila* heart. Existing collections of lethal transposon insertion mutants have also been screened through. As an alternative to using point mutations, collections of overlapping deficiencies, which have been assembled by the Bloomington Stock Center and uncover 98.4% of the euchromatic genome [4], have been used. Here, the ultimate goal was to pinpoint the individual genes responsible for the observed heart phenotypes of homozygous deficiency embryos. RNA interference screens have also been used successfully to identify new genes that are required for normal heart development. Each of these screening methods (Table 1) has its specific advantages and disadvantages. Chemical saturation mutagenesis provides the most unbiased approach, but the identification of the affected genes takes longer as compared to the other methods. In screens with transposon-induced mutations, the affected genes can easily be pinpointed through the insertion sites. However, due to the insertional bias of most transposons, only a fraction of all genes can be screened, and frequently the mutations are weak alleles as insertions into protein coding regions are quite rare. With deficiency screens, null mutant phenotypes are obtained and the approximate locations of the genes responsible for them are already known. However, early embryonic phenotypes caused by the absence of other deleted genes can often obscure the role of genes acting specifically upon the heart. RNAi screens provide the great advantage that the identity of the targeted gene is known from the outset. However, with this approach, important genes can be missed because of inefficient knock-downs. False-positives can arise from off-target effects and other artifacts [5] that need to be controlled for in follow-up experiments (e.g., by classical genetics or targeted tissue-specific protein degradation, deGradFP [6]). Although in principle each of these methods has the potential to cover almost the entire genome, in practice none the screens performed to date has truly interrogated all genes of the genome. The mutational screens have focused on the two major autosomes and the RNAi screens have been limited by the work-load and/or the existing lines for inducible RNAi. Nevertheless, all of these screens have uncovered interesting new genes and pathways that regulate heart development.

### 2.1. Screens with Deficiency Collections and EMS-Induced Mutations 

Tao *et al.* [7] performed a deficiency screen for the second chromosome using a deficiency collection that covered ~95% of this chromosome, which corresponds to a coverage of ~38% of the genome. Heart phenotypes were scored in live homozygous deficiency embryos, into which either *tup-GFP* (a cardioblast-specific reporter driven by a *tailup* enhancer) or *Hand-GFP* (a reporter for both cardioblasts, pericardial cells, and the hematopoietic lymph glands [Figure 1] driven by an enhancer of the *Hand* gene) had been introduced as specific heart markers. The observed phenotypes included an altered morphology and assembly of the heart tube as well as decreased and increased numbers of heart cells. The fact that ~60% of the tested regions caused heart phenotypes indicates that many of the deficiency intervals included genes that either affect heart development indirectly, perhaps because they are already required for early steps during embryogenesis that precede heart development. Yet, others may have ubiquitous functions needed in most or all cells of the embryo, including those of the heart. However, in several deficiency intervals, individual genes were identified that have more specific roles in various aspects of cardiogenesis. These include the lipid phosphatase encoding gene *wunen* and its ortholog *wunen2*, which were found to be required for the adherence of the pericardial cells to the cardioblast tube. Both genes are expressed prominently in migrating cardioblasts and were subsequently shown to be required additionally for the reliable joining of the contralateral cardioblast rows at the dorsal midline [25]. Other examples include a *troponin C*-related gene, *tina-1*, which is expressed in posterior cardiomyocytes and appears to be needed for the correct assembly of the posterior end of the heart tube, and *CSN4*, which encodes a component of the COP9 signalosome that has pleiotropic functions. In the heart it is required for establishing the correct ratio of ostial *versus* working cardiomyocytes [7]. The exact roles of these latter genes and several others from this screen still need to be examined in follow-up experiments.

In another screen, *ca.* 3000 homozygous lethal mutation lines (including P-element or PiggyBac insertions, EMS mutants and deficiencies) from the Bloomington Stock Center were screened with the *Hand-GFP* reporter for late embryonic heart defects [8]. The full data from the screen have not been published, but one class of mutants that display a dissociation of the pericardial cells from the cardioblasts at late stages of cardiogenesis, collectively called “broken hearted” (“bro”) mutants, have been examined in detail. One of these bro mutants disrupted the trimeric G protein subunit Gγ1 and three others affected different enzymes in the mevalonate pathway that are required for geranylgeranylation of the C-terminal CAAX motif of Gγ1. These and additional data suggested that proper membrane anchoring and functioning of the trimeric G protein consisting of Gγ1, Gβ13F, and G-oα47A within the cardioblasts is a prerequisite for the stable attachment of the pericardial cells to the cardioblasts [8,26]. Furthermore, another bro gene was identified as *neurexin IV* (*nrxIV*), which encodes a transmembrane protein normally found in septate junctions. Additional follow-up experiments led to the conclusion that trimeric G protein signaling is required for regulating the proper cellular localization of NrxIV and a number of other proteins that are part of septate junctions in other tissue contexts (even though septate junctions *per se* are not found in cardioblasts). In part, interactions of the extracellular domains of Nrx IV and perhaps other septate junction proteins at the lateral (basal) “pericardial adherent domains” of the cardioblasts with extracellular matrix components such as the collagen α-1(IV) related protein Pericardin may mediate stable pericardial/cardial cell adhesions [26]. Thus, this screen identified several components in a common pathway that is crucial for maintaining the integrity of the cardiac tube and made it possible to characterize this pathway in depth.

A chemical saturation screen for the 2nd chromosome was performed in embryos in the presence of the *tinman-GFP* reporter (*tinCΔ4-GFP*) as a cardioblast marker and an RFP reporter (*org-1HN18-RFP*) for the alary muscles, which are segmental muscle fibers that anchor the heart to the epidermis (Figure 1) [9,10]. Apart from these heart structures, *org-1-RFP* additionally marked several other somatic muscles and a third reporter, *HLH54Fb-RFP*, allowed simultaneous screening for mutants with longitudinal gut muscle defects [27]. Among the 469 homozygous lethal lines with defects in embryonic somatic muscles, gut muscles, or the heart, there were 313 lines with interesting heart defects that featured either decreased or increased numbers of heart cells or various types of morphological abnormalities of the heart tube [10]. Published reports on heart-related genes identified in this screen describe the functions of the FGF8 encoding genes *pyramus* and *thisbe* during cardiogenesis and the migration of longitudinal gut muscle progenitors [27]. In addition, the functions of two genes encoding ECM proteins, namely the laminin β protein LamininB1 (LanB1) and the collagen IV α1 protein Cg25C, in the stable attachment of the alary muscles and pericardial cells to the myocardial tube were reported [9]. The similarities of the *LanB1* and *Cg25C* phenotypes with those of the “broken hearted” class of mutants further support a functional connection between septate junction proteins and the correct assembly of the cardiac ECM, and underline the key role of the cardiac ECM for the integrity of the heart tube.

Yet, another mutation with an interesting connection to the cardiac ECM was reported as a result of a genetic screen of a collection of pupal lethal EMS mutants that also employed *Hand-GFP* as a marker for the heart tube [11,28]. The mutation in a gene termed *lonely heart* (*loh*) again displays a broken hearted phenotype with pericardial cells and alary muscles becoming detached from the myocardial tube and an altered ultrastructure of the cardioblasts. *loh* encodes a heart-specific ADAMTS-like protein of the cardiac ECM and was shown to be required as a receptor for incorporating the atypical collagen Pericardin into this ECM [11].

In sum, while only a fraction of the data from these genetic screens for heart mutants have been fully analyzed and published to date, it is notable that many of the genes identified so far are involved in the assembly and stabilization of a specific ECM around the heart. This cardiac ECM is important for the integrity of the heart tube and particularly for the stable attachment of the pericardial cells and alary muscles to the myocardial cells.

### 2.2. Screens with RNA Interference (RNAi)

In a first systematic RNAi screen for heart phenotypes, double-stranded RNAs against over 5800 genes (~40% of the total number of known and predicted genes) were injected into preblastoderm stage embryos. In the initial round of screening, three dsRNAs were pooled for each batch of injections. The injected embryos carried a cardioblast-specific *Mef2-lacZ* reporter, which was used for analyzing the hearts of late stage embryos via X-gal stainings [12]. *Ca.* 130 genes with potential roles in heart development were identified by this approach. Although these include genes with early and broad developmental functions such as segmentation genes, dorsoventral axis regulators, and cell cycle control genes, the fact that also several known cardiogenic regulators were identified provided validation for the approach. One of the novel genes characterized in some more depth, termed *simjiang* (*simj*), is required for the formation of a subset of cardiac progenitors, including two of the four Tinman^+^ cardioblasts within each hemisegment and certain pericardial cells. The mammalian counterpart of Simj, p66, is a constituent of the NuRD histone deacetylase complex. More recently, it was shown that Simj/p66, which is present in all cells including the heart, is involved in the repression of Wg target genes [29]. It is unclear whether this property underlies the knock-down phenotype in the *Drosophila* heart because, due to the sequential roles of the Wg pathway in the mesoderm, its derepression can either lead to an increase or a decrease of the number of heart cells [30]. Of note, a recent study has described specific roles of the NuRD repressor complex in vertebrate heart development [31].

The generation of genome-wide transgenic RNAi libraries made it possible to perform RNAi screens by tissue-specific knock-downs with the binary UAS/GAL4 system [32,33]. In one such screen with the heart by Neely *et al.* [13], over 7000 evolutionarily-conserved genes were knocked down with a cardioblast-specific *tinman-GAL4* driver specifically within the myocardial tube. The screen used shortened life spans of adult flies as selection criteria, as it is known that the absence of a functional heart does not result in strict lethality in *Drosophila* [34]. In addition, the screen was performed at both 25 °C and 29 °C, the rationale for the higher temperature being that expression levels of dsRNAs are increased and the heart is sensitized due to the faster heartbeat. Thus, the knock-down efficiency is increased and additional genes should be uncovered at 29 °C, including those involved in functional aspects of the heart. 465 candidates were selected from the 25 °C screen and close to 500 additional candidates from the 29 °C screen. Among these candidates, there should be genes that are specifically involved in regulating heart functions. However, both candidate pools are also expected to include many genes that affect heart function indirectly, either through their roles in regulating cardiogenesis or through their participation in basic cellular processes within cardioblasts that are shared with other cells. Making a clear distinction among these classes of genes would be a major effort and has not been attempted yet. One group of candidate genes that were selected for detailed examination encode components of the CCR4-Not protein complex, which is known to fulfill a myriad of functions in transcriptional and posttranscriptional gene expression [35]. Knock-downs of these genes with the *Hand-GAL4* driver appears to affect heart function at least in part by disrupting the normal formation of the adult ventral longitudinal heart muscles [13] (Figure 1B), perhaps as a result of perturbing the expression of various genes that regulate their development [36].

## 3. Analyses of the Transcriptome and Proteome of the *Drosophila* Heart

The analysis of the transcriptome and proteome of the heart under various conditions has the potential to generate a large trove of new information (Table 1). This could include, (1) the discovery of new regulators of heart development; (2) the delineation of dynamic changes of gene expression, alternative splicing, and protein contents during heart development; (3) the determination of the global changes in these events upon interfering with the function of critical regulators of cardiogenesis. Due to the minute size of the embryonic heart, so far these approaches have been limited to the cardiac tube of larvae, pupae, and adult flies, which can be isolated manually and pooled from large numbers of animals. It should be noted that, in addition to the myocardial cells of the heart tube and pericardial cells, a significant fraction of this material from adults consists of the ventral longitudinal muscles that are tightly associated with the ventral surface of the heart tube and share many features with skeletal muscles [37] (Figure 1B). The utilization of more refined techniques such as fluorescent cell sorting (FACS) of genetically-marked Drosophila heart cells [38,39], Thiouracil (TU) tagging [40] or targeted DNA adenine methyltransferase identification (TaDa [41]) should make it possible in the near future to extend studies of this type to embryonic hearts as well.

### 3.1. Whole Genome Expression Profiling during Remodeling of the Larval into the Adult Cardiac Tube

Aiming to correlate the known morphological changes during heart remodeling with changes in global gene expression, Zeitouni *et al.* [14] have profiled the transcriptomes of developing adult cardiac tubes at successive time points during metamorphosis on microarrays. Six time points within a ~27 h period covering the major events between the destruction of portions of the larval heart and the differentiation of the adult heart were used. Among the >4800 expressed genes, 1660 showed significant differential expression (>1.8 fold) between time points. These genes were grouped into 13 clusters with joint expression profiles, in which sets of genes were defined as progressively repressed or activated, or transiently activated or repressed, during the remodeling process. Among the genes that initially are over-active and then progressively became repressed by the second half of the remodeling, genes involved in proteolysis, ion and vesicle transport, cell death and histolysis, muscle contraction, and histone modification were overrepresented. These dynamic changes likely reflect the events in which the two posterior segments of the larval cardiac tube are histolyzed and the remaining portions of the heart partially (or for some alary muscles completely) dedifferentiate during this period (Figure 1). During the middle of the remodeling process, the gene sets that are activated temporarily are enriched for genes that encode cell surface receptors, signaling proteins, and proteins involved in myoblast fusion and muscle development. Likely, these reflect genes that are required for reinitiation of cardiogenesis of the adult myocardial tube and myogenesis of the associated ventral longitudinal heart muscles. On the other hand, metabolic genes are downregulated, which correlates with the absence of contractility during this period. Finally, among the gene sets that become activated only during the second half of the remodeling process, genes encoding proteins involved in mesoderm development, muscle contraction, energy and lipid metabolism, and cell matrix adhesion are overrepresented. This can be explained by the processes of morphogenesis and functional differentiation of muscle tissues during the formation of the adult heart during this period. The entire remodeling process is orchestrated by an ecdysone signaling circuit and the observed successive activation of ecdysone response genes is in line with analogous events occurring during remodeling of other tissues during metamorphosis.

For functional verification of the results, the study focused on several signaling pathway components with dynamic expression during the remodeling process. For example, gene transcription for the Wnt receptor Frizzled (Fz), the glypican co-receptor Dally, and the ligand Wnt4 is upregulated transiently during the middle of the remodeling period. Experiments with forced inhibition and overactivation of the pathway in the myocardial tube led to the conclusion that, in the normal situation, the Wnt signaling pathway serves to suppress terminal chamber (A5) identities in the actual heart (Figure 1B). The adult heart arises from the posterior aorta of the larval heart within abdominal segments A1 to A4 (Figure 1). Whether Wnt4 or Wingless (Wg) is the crucial signal in this process has not been established. Wnt signaling is additionally required for the formation of the segmental ostia (inflow tracts) in the adult heart, which fits with earlier observations that Wg is expressed specifically in ostial cells of the larval heart chamber [42]. Genes encoding components of another signaling pathway, namely for the PDGF-VEGF-related receptor tyrosine kinase Pvr and one of its ligands, Pvf2, became activated (permanently for *Pvr* and transiently for *Pvf2*) during the middle of the remodeling process. It was found that *Pvr* expression specifically occurs within the cardiac valve cells, which are formed from single pairs of myocardial cells within each abdominal segment from A2 to A4 (Figure 1B). Experiments with forced blockage and ectopic activation of the pathway led to the conclusion that Pvr signaling is necessary and to some degree sufficient for the formation of adult cardiac valves from cardiomyocytes. Additionally, the roles of components of the FGF and Notch signaling pathways were examined, which showed dynamic expression patterns of the genes for their various ligands, receptors, and inhibitors, respectively. These two pathways were found to have important roles during the formation of the ventral longitudinal muscle fibers of the adult heart (Figure 1B). The role of FGF signaling during this process, which involves transdifferentiation of anterior larval alary muscles, recently has been described in more detail [36]. Hence, this approach has yielded novel insights into the global changes and specific regulators of larval-to-adult heart remodeling.

A follow-up study used an *in silico* approach to determine whether different genes within a particular expression cluster are controlled by functionally-related *cis* regulatory modules (CRMs) [15]. In particular, genes from a cluster with very late upregulation during heart remodeling featured high enrichment scores for position weight matrices (PWMs) of nuclear receptors, which are mediators of the ecdysone response, within their −5 kb and intron sequences. When fragments from six genes, which contained motifs of this type with the highest scores and surrounding conserved sequences, were tested by reporter gene analysis *in vivo*, their temporal enhancer activity closely matched the dynamic expression of the corresponding genes from the microarray data. *In vivo* data with mutated binding motifs as well as knock-downs and forced expression of candidates for relevant nuclear receptors indicated the involvement of both positive and negative inputs by these regulators. Notably, while reflecting the correct temporal profile, the identified enhancers were active broadly but not in the heart [15]. Hence, the obtained information provides insight only into mechanisms of temporal regulation but not into tissue specificity, which is likely controlled by yet unidentified CRMs that may combine analogous temporal control sequences with motifs for heart-specific factors.

### 3.2. Determination of the Proteome of the Adult Fly Heart

Cataloguing the protein composition of the heart can be an alternative approach to gain insight into the regulation and progression of heart differentiation. Although the small size of the *Drosophila* heart is currently a limiting factor, a recent study analyzed the proteome of hand-dissected heart tubes from adult flies for this purpose and identified ~1200 protein clusters and associated genes [16]. Seventy-one percent of these were also represented in the transcriptomes of metamorphosing hearts as determined by Zeitouni *et al.* [14], and another 24% were present below the threshold chosen in these transcriptomes, perhaps because their expression is only just beginning during the pre-adult stages. *Ca.* 15% of the ~500 genes from the screen by Neely *et al.* [13] that shortened life spans upon heart-specific knock-down specifically at 29 °C were represented in the proteome. In terms of their abundance, myofilament proteins (particularly myosin), cytoskeletal proteins, mitochondrial proteins (including those involved in energy metabolism), proteins involved in protein synthesis (including ribosomal proteins), and proteins of the basal lamina made the largest contributions, as might be expected from muscle tissues. Accordingly, the distribution of different molecular and functional classes of proteins found in the *Drosophila* heart was similar to that found previously in proteomic analyses of the mouse heart [43]. Although the coverage of proteins with low abundance, which likely include interesting regulators of differentiation, was insufficient in this exploratory proteomics screen, future improvements of the sensitivity of detection could make this a promising avenue for studying the development and differentiation of the *Drosophila* heart.

### 3.3. Differential Profiling of Global Gene Expression in Mutant *Versus* Wild Type Heart

Differential profiling of the transcriptomes in wild type hearts *versus* hearts that carry a mutation in a particular regulatory gene can be used to characterize the molecular responses that normally are set in motion downstream of the respective regulator. This rationale has been used in a first attempt to define the downstream events that require the cardiac bHLH transcription factor Hand in the developing heart [17]. Hand is expressed in both cardial and pericardial cells throughout development [44] (Figure 1). Whereas Hand does not have any overt functions during embryonic cardiogenesis and the formation of the larval heart, it plays an important role during later stages, particularly during metamorphosis when it is required for generating an adult heart with a normal morphology and myofibrillar organization of the cardiomyocytes [45].

The transcriptomes from wild type *versus Hand* mutant hearts, which were isolated from 3rd instar larvae and tested on genomic microarrays, were compared to each other. 385 genes showed diminished expression and 160 genes had increased expression levels in the mutants. Verification by RT-PCR or Northerns was performed for the subset of genes within these two groups that had also been found in the above-described screens of Zeitouni *et al.* [14], Neely *et al.* [13], and Cammarato *et al.* [16]. The microarray results were confirmed in ~40% of the cases (17 genes) and included the genes for Muscle-specific protein 300 (Msp-300), Akirin, Zasp52, and Multiplexin. These proteins are known to be important for different aspects of the architecture or function of muscles [46,47,48] and, in the case of Multiplexin, of the heart [49]. The expression of these genes was reduced but not abrogated in *Hand* mutants, which was taken as an indication that Hand may act as a modulator rather than an essential trigger of transcriptional outputs. However, it needs to be established whether any of these genes are direct transcriptional targets of Hand. In addition, it is likely that the transcriptional responses to Hand are more pronounced during remodeling in the pupal heart, when its functional absence results in the most conspicuous heart phenotype.

A more extensive analysis of this type of transcriptomics screen was performed by Ahmad *et al.* (2012) [18], who aimed at identifying new genes expressed in the cardiogenic mesoderm (CM) and heart. The basic strategy was to screen for transcripts that are enriched in the dorsal mesoderm as compared to the entire mesoderm, are underrepresented in the mesoderm of mutants that are known to have reduced cardiac tissues, and are overrepresented in the mesoderm upon genetic manipulations known to exhibit excessive amounts of cardiac tissues. Flow cytometric purification of dorsal mesodermal wild type cells marked with a Dpp-responsive *tinman* reporter and of cells from the entire mesoderm from the various mutant backgrounds was used to generate the sources used for these cross-comparisons. The statistical meta-analysis of the data was fitted to a training set of 40 genes known to be expressed in the CM. From 136 randomly selected genes that ranked among the top 400 candidates of the obtained list and did not include any genes from the training set, 70 were expressed in the CM and/or heart (as well as in various other tissues) based upon *in situ* hybridizations. In a follow-up study [19], an additional 50 genes with heart cell expression were verified (although discrepancies of the expression annotations with the data from BDGP [50] still need to be resolved). Hence, this approach yielded numerous new candidates for genes with possible functions during heart development. To test this expectation, two of the genes from this list, one encoding the Forkhead domain transcription factor Jumeau (Jumu) and the other encoding Polo, a kinase regulating various mitotic events, were chosen for functional analyses. Based on *in situ* hybridizations, the transcripts of both genes are present, although not highly enriched, in the CM of wild type embryos. The authors could show that *jumu*, in cooperation with the Forkhead domain gene *Ches-1-like*, is required upstream of *polo* for ensuring proper symmetric and asymmetric divisions of heart progenitors in a faithful manner [18].

## 4. Genome-Wide Searches for Binding Sites and Target Genes of Cardiogenic Transcription Factors

The developmental roles of several cardiogenic transcription factors (TFs) have been well established during recent years. These include the homeodomain protein Tinman (Tin), the GATA factor Pannier (Pnr), the Dorsocross T-box factors (Doc1, Doc2, Doc3), the T-box factors Midline (Mid, aka Nmr2) and H15 (aka Nmr1), the LIM homeodomain protein Tailup (Tup), the COUP-TFII factor Seven-up (Svp), and the bHLH factor Hand. The position of these factors and their genes within a regulatory network of heart development has been defined in much detail [1]. It is also known that many of these cardiogenic genes, as well as the gene for the myogenic differentiation factor Mef2, are themselves direct targets of cardiogenic factors within this regulatory network [51,52,53,54,55,56,57]. However, much less has been known about the direct targets of these cardiogenic factors that ultimately carry out the functions of these regulators to provide heart cells with their specific shapes, arrangements, and functional properties. Through candidate approaches, a few of such “realizator genes” have been defined as direct targets of cardiogenic TFs, such as *β3-tubulin*, *Sulfonylurea receptor* (*Sur*; encoding a modulator of the inwardly rectifying K+ channels), and *Toll* (*Tl*, encoding a transmembrane receptor) [58,59,60,61]. For a complete understanding of the *cis*-regulatory networks driving cardiogenesis, ideally the full complement of direct targets that are either activated or repressed by each of the cardiogenic factors needs to be defined. With the advent of genomics, this undertaking can now be attempted by probing the genome for *in vivo* binding sites of these factors via immunoprecipitation of bound chromatin followed by microarray assays (“ChIP-chip”) or massive parallel sequencing (“ChIP-seq”). Because the fully developed heart contributes less than 1% of the nuclei to the embryo, so far these approaches have been performed with earlier stages when the cardiogenic mesoderm makes up a larger fraction of the embryo and better signal-to-noise ratios can be obtained (Table 1). In future experiments, enrichment of heart cells or nuclei prior to immunoprecipitation may circumvent this limitation (e.g., BiTS-ChIP) [62,63]. In addition, methods employing inducible GFP fusion proteins have recently been developed that allow tissue specific ChIP-seq even for factors that are expressed in several different tissues (CAST-ChIP [64]). This latter method, as well as the rapidly increasing availability of fly lines with GFP-tagged genes in the native context [65,66], will also permit ChIP-seq approaches with factors for which antibodies are not available.

The first screen for *in vivo* Tin binding sites was done by ChIP-chip on microarrays that covered ~50% of the *Drosophila* genome with overlapping 3 kb fragments [20]. Three consecutive time periods were probed, spanning the stages of gastrulation up until the formation of heart primordia, and 481 unique Tinman-bound regions were identified that were associated with 260 putative target genes. For functional verification of selected genes, the study focused on genes that are activated in the somatic mesoderm, potentially being downstream of the early, broad expression of Tin in the whole mesoderm. However, several *in vivo* binding sites were located within previously defined enhancers with activity in the developing heart, including those of *tin* itself (autoregulation together with activated Smads [67]), *Mef2* (in cardioblasts [51]), *even-skipped* (*eve*; in pericardial cells [68]), and *seven-up* (*svp*; in developing ostial cardioblasts [54]). Other binding sites were present at untested sequences near known cardiac regulatory genes, including *pannier* (*pnr*), *midline* (*mid*), *apontic* (*apt*), and *odd-skipped* (*odd*). These data also demonstrated temporal specificities in binding site occupancy and, in general, validated the approach.

Two more recent studies performed by Junion *et al.* [21] and Jin *et al.* [23] directed their main emphasis towards the *in vivo* binding and regulatory function of cardiogenic factors at enhancers within heart primordia and developing heart cells. Because of the known key roles of Tin, Doc, and Pnr during early cardiogenesis [1] and the previous finding that the combined action of all three of these factors is needed for efficient induction of myocardial fates in ectopic expression experiments [69], these three factors were analyzed together at the genomic and/or enhancer level in these two studies. Additionally, Junion *et al.* included the nuclear effectors of Dpp (*i.e.*, the Smad protein Mad) and Wingless (dTCF/Pangolin) in their analysis, as it is known that heart progenitors are induced at the intersection of dorsal Dpp and striped Wingless signals [1]. The global binding data in the two studies were generated via ChIP-chip on the same type of *Drosophila* high density tiling arrays (Affymetrics) and were performed at two similar developmental stages. The early stage (4–6 h [21] or 3–5.5 h [23] after egg laying) corresponded to the stages of mesodermal spreading and subdivision into individual primordia, including the cardiogenic mesoderm, whereas the later stage (6–8 h [21] or 5–8 h [23]) spanned the period from mesoderm patterning until specification of heart progenitors.

The investigation by Junion *et al.* [21] was more comprehensive, as it determined global binding site occupancies of four factors, Pnr, Doc, Mad, and dTCF experimentally, in addition to incorporating the binding data for Tin from a previous study by Zinzen *et al.* [22]. Clustered binding sites of any combination of these factors, which were candidates for *cis*-regulatory modules (CRMs), occurred at >11,000 positions in the genome, and ~4000 among these included Tin binding sites. The putative CRMs with Tin binding sites could be classified into different types, with one class showing enrichment for all five transcription factors (TFs), a second showing only Tin occupancy, and four others showing elevated occupancy of Tin plus one of the other TFs but medium or low binding of the remainder. Of note, the most frequent class by far was the one with occupancy by all five factors (“All TFs” class), which was followed in frequency by the classes “Tin only” and “Tin + Pnr”. By contrast, when Tin-negative regions were analyzed, no significant enrichment for co-occupancy of any of the other four TFs was detected. A possible explanation could be that Tin is the only one among this TF collection that is expressed or active almost exclusively in the mesoderm. Hence, the expression and activity domains of the five TFs only overlap in mesodermal areas. In these areas, the TFs tend to become co-recruited to regulatory regions, perhaps in a cooperative manner and because mesodermal enhancers are in an open, accessible configuration and include binding motifs for several or all of these TFs. *Ca.* 50%–60% of the regions occupied by all 5 TFs did indeed contain close matches to binding motifs for Tin, Pnr or Doc, while the prevalence of dTCF and Mad binding motifs was lower. It was proposed that cooperative interactions among cardiogenic TFs, and potentially protein-protein interactions with chromatin activators such as CBP/p300, could be involved in co-recruiting even those TFs that lack high affinity (or any) binding sites at a given cardiogenic enhancer [21].

To test the significance of joint TF occupancy, 55 regions with average lengths of 550 bp were examined for enhancer activity *in vivo*. The large majority was indeed active in areas of the mesoderm (apart from various additional tissues). Notably, >40% of the “All TFs” class and the “Tin + TCF” class included activity in the cardiogenic mesoderm, while the visceral mesoderm was the second most frequent mesodermal tissue with enhancer activity in these two classes (~25%) (As some of these expression data were revised in a subsequent study [19], the actual percentages are somewhat lower). The cardiac enhancers from the “All TFs” class were heterogeneous with respect to their specific composition, spatial arrangements, and orientations of TF binding motifs, thus lacking a consistent “binding site grammar”. This suggests a functional flexibility of jointly bound TFs, but in part it presumably also reflects the spatial and temporal heterogeneities of the enhancer activities both in the cardiogenic mesoderm itself and in various additional tissues of the embryos. In a cell culture assay, three tested enhancers from the “All TFs” class most strongly responded to a combination of all five factors, although the largest contribution to the activity was provided by the combination of Tin, Pnr, and Doc.

Whereas the effects of combinatorial binding of cardiogenic TFs on promoting cardiac activities to CRMs are intuitive and in agreement with previous genetic data, the result that ~25% of the tested regions in the “All TFs” class provided enhancer activities in the visceral mesoderm seemed counterintuitive. The primordia of the visceral and cardiac mesoderm are arranged in an alternating fashion along the anterior-posterior axis in the dorsal mesoderm. Although the visceral mesoderm primordia share the presence of Tin and active Smads with the cardiogenic primordia, they lack Doc, Pnr, and active dTCF. The observed binding of Doc, Pnr and dTCF, in addition to Tin and Mad, therefore should reflect the inactive status of these visceral mesodermal enhancers within cells of the cardiogenic primordia and not their active status in the visceral mesoderm. Because, as shown with cardiogenic enhancers, these combined TFs normally promote enhancer activity, the inactive status of enhancers bound by these TFs within the cardiogenic primordia implies the additional binding of one or several repressors. A candidate for such a repressor, namely the Forkhead domain protein Sloppy paired (Slp), had been identified in the course of the functional dissection of the visceral mesoderm enhancer of *bagpipe* (*bap*) [70]. *slp* is induced by Wg in striped mesodermal domains encompassing the prospective cardiogenic primordia, where it binds to the *bap* enhancer to repress it even though the activating factors Tin and Smads are also bound. Indeed, ChIP-chip for Slp demonstrated that enhancers within the “All TFs” class that displayed visceral mesoderm activity on average were occupied more highly by Slp than those active in the cardiogenic mesoderm. When the Forkhead domain motifs, *i.e.*, the presumed Slp binding sites, were mutated, two out of three tested enhancers now indeed showed significant derepression in the cardiogenic mesoderm (and inexplicably, also in the somatic mesoderm). At the same time, visceral mesoderm activity disappeared, which can be explained by the usage of the same motif by the forkhead domain factor Biniou (Bin), a major positive regulator in the visceral mesoderm [71,72]. Taking these observations together, the authors proposed that numerous cardiogenic and visceral mesodermal enhancers share the feature of being bound by a cardiogenic TF collective, but that the function of these TFs is “dormant” in the visceral mesodermal enhancers within the cardiogenic mesoderm due to the co-bound Slp repressor. They speculated that this shared feature might represent a relic from a common evolutionary origin of the two mesodermal tissues that has been proposed by some authors [73]. Because only few visceral mesodermal enhancers were tested *in vivo* to back up these models and the assays were limited to the Forkhead domain binding motifs, the validity of these interesting proposals needs to be further confirmed.

Jin *et al.* detected >2500 genomic Tin-associated sites in the early embryonic stages. This compared with ~1150 Tin sites in the corresponding stages found by Zinzen *et al.* [22] (using the same cut-off), of which ~870 were present in both sets. Both studies found ~1000 Tin sites in the equivalent later stages, with over 60% being common to both sets. These comparisons underscored that the results with this methodology are quite reproducible even under different lab conditions. Interestingly, 60%–85% of the sites occupied in the later stages were also occupied in the early stages. The differences were due to the disappearance of sites occupied early and the appearance of newly occupied sites in the later stages, thus showing dynamic binding to a subset of sites. As might be expected, the Gene Ontology (GO) terms “heart development’’, ‘‘mesoderm development’’ and ‘‘gastrulation’’ were the most strongly overrepresented developmental terms for the genes flanking the binding sites at both stages, although for unknown reasons “imaginal disc” development was also high on the list. In molecular terms, genes encoding transcriptional regulators were clearly overrepresented, which strengthens the notion that Tin fulfills a key role within a transcriptional network of mesodermal and heart development.

*De novo* motif searches identified the known Tin-binding motif as the most strongly enriched one among the Tin-bound sequences, and among 6-mers, a GATA factor motif likely connected with binding of Pnr was the most strongly enriched motif. T-box (including Doc) binding motifs, although not retrieved in *de novo* motif searches, did occur in a significant number of Tin-bound sequences. These data reinforced the findings of frequent co-occupancy by Tin, Pnr, and Doc from Junion *et al.* [21], which in many cases is explained by the clustered presence of the binding motifs for these factors. As previous data had shown that high occupancy levels correlated with functional binding of TFs [74] while low occupancy sequences mostly do not correspond to enhancers [75], Jin *et al.* [23] ranked the Tin-bound sequences according to the strength of Tin association and further analysis focused on the sequences and their associated genes ranked highly on this list. Indeed, most of the known Tin target or downstream genes were found to be associated with the top ~250 sequences most highly occupied by Tin.

Among the 51 *in vivo*-tested sequences (with 1400 bp average lengths) that displayed high Tin occupancy during either or both of the developmental time windows and contained at least one high-scoring Tin binding motif, 76% were active as enhancers in areas of the mesoderm that overlapped with Tin expression. Forty-four percent of them were active in the dorsal or cardiogenic mesoderm and/or in the developing heart (and some of these additionally in the somatic or visceral mesoderm). These data indicate that the selection of sequences highly occupied by Tin that contain Tin motif(s) and the selection of sequences co-occupied by several cardiogenic TFs as in [21] have about the same success rate in identifying *cis*-regulatory modules that are active in developing heart tissues. The activity of these CRMs in cardiogenic tissues and the heart was critically dependent on Tin activity, as shown by loss of activity of cardiogenic mesoderm enhancers in *tin* null mutant backgrounds and of heart enhancers in embryos lacking Tin specifically in the heart [23].

Tin occupancy and *tin*-dependency of the cardiac activity of the identified enhancers provided strong indication, but not proof, that these enhancers and their associated genes are direct targets of Tin. More stringent proof for being a direct target of Tin, or of any of the other cardiogenic TFs that show occupancy *in vivo*, can be obtained by additionally showing that the respective binding motifs for these TFs are required for normal enhancer activity. Six enhancers with lengths of 600–800 bp were selected for these tests with mutated binding motifs. One enhancer (from the *Egfr* locus) also contained GATA motifs in addition to Tin-binding motifs, and each of the other five (from the loci of *lin-28*, *mid*, *RhoL*, *tup*, and *unc-5*) contained all three binding motifs for Tin, GATA-factors, and T-box-factors. Consistent with the observations made by Junion *et al.* [21], the numbers and specific arrangements of these motifs were highly heterogeneous among these enhancers. According to the global binding data from Junion *et al.* [21], the CRMs of *mid* and *tup* were only occupied by Tin (during the stages assayed), whereas the other four enhancers were occupied by all three TFs, Tin, Doc, and Pnr.

Interestingly, each of these CRMs turned out to differ with respect to the specific requirements for the binding motifs for the individual TFs. Although the *tup* enhancer contained all three binding motifs, it only required the Tin motifs for its activity in the cardiogenic mesoderm, a result that matched its exclusive occupancy by Tin *in vivo*. The *mid* enhancer, which was also reported to be occupied only by Tin, was fully dependent on its Tin motifs as well, not dependent on its GATA motifs, but partially dependent on its T-box-binding motifs. The latter result could either be due to low-level binding of Doc (below the cut-off used in [21]) or to binding of the T-box factor Mid in an autoregulatory fashion. The *Egfr* and the *lin-28* CRMs required both the Tin and the GATA motifs, but the T-box motifs in the *lin-28* CRM were not essential. Strikingly, the CRM associated with *RhoL* neither required the Tin motifs nor the GATA motifs, but in this case, the T-box motifs, presumably via Doc binding, were essential for its activity in the cardiogenic mesoderm. Besides non-functional binding of a TF, the lack of requirement for particular binding motifs of individual TFs shown for some of these CRMs could also be explained by functional redundancy among different TFs bound to the same CRMs. Evidence for functional redundancy among cardiogenic TFs was obtained with the *unc-5* CRM, which is active both in cardioblasts and in pericardial cells. In this CRM, mutation neither of the Tin motifs nor of the GATA or T-box motifs affected its activity in the two types of heart cells, and only simultaneous mutation of all three types of motifs completely extinguished its activity in the cardioblasts (but not in the pericardial cells, where it therefore relies on yet undefined factors). In a recent study with an overlapping fragment that lacked some of the Tin, GATA and T-box motifs assayed by Jin *et al.* and showed weaker activity in cardioblasts, the requirement for the remaining Tin sites did become apparent [76]. Therefore, although all three factors are bound *in vivo*, in this case binding of a sufficient number of molecules of either Tin, Pnr, or Doc seems to be enough for full activation, thus indicating a modus of mass action.

In sum, these ChIP-based screens for global occupancies of DNA sequences by cardiogenic factors have expanded our understanding of regulatory networks during heart development significantly. In particular, they uncovered genome-wide candidates for CRMs and their associated genes that are regulated by these factors and follow-up experiments have defined many new examples of their target enhancers and target genes. It is important to recognize that not all sequences occupied *in vivo* by these factors and their associated genes are biologically relevant targets of these factors, and that the proportion of such functional targets among the globally-occupied ones is difficult to determine. In some cases, binding of a cardiogenic factor may have modest influences on the regulation of an associated gene that are not essential for normal heart development. Nevertheless, they may increase the robustness of the process and therefore provide a selective advantage. In other cases, binding may be due to “passive hitchhiking” of a factor, for example because its binding motif happens to be present in a CRM that was opened in terms of its chromatin structure by other tissue-specific TFs, or because of protein interactions with such TFs. In some of these situations, binding of a cardiogenic factor might not influence the enhancer activity of the CRM due to the absence of proper co-factors, or if it does, the modulation of the expression of the associated gene might be inconsequential for heart development or its robustness. In addition, several examples were found where the neighboring genes on either side of a CRM with activity in the developing heart were not expressed in a similar heart-specific fashion ([19], Hong Jin and M.F., unpublished observations). In these cases, methods such as tissue-specific chromosome conformation capture [77] may be necessary to define the real target genes that are controlled by such enhancers, potentially at great distances. These examples illustrate that approaches at the genomic level, although being extremely informative in providing a global picture of events such as heart development and in providing new hypotheses, need to be followed up by appropriate experiments to test these hypotheses in order to obtain new conclusive insights into the process.

## 5. Computational Analyses Utilizing Genomic Data in *Drosophila* Cardiogenesis

The increasing numbers of various types of genomic datasets that are becoming available on *Drosophila* cardiogenesis require computational approaches in order to deliver their full potential. For example, with a collection of cardiac-specific enhancers at hand, it is now possible to ask the question of whether these enhancers are enriched for any sequence motifs in addition to those of the binding motifs of the known cardiogenic factors. Computational approaches of machine learning can be used for achieving this goal and, likewise, for identifying additional cardiac enhancers based on classifiers derived from already known sets of enhancers (Table 1) [78]. Using a classifier derived from a training set of 24 cardiac enhancers *versus* 37 enhancers active in other mesodermal tissues, Jin *et al.* [23] identified a motif with a high predictive value for cardiac enhancers and termed it “Cardiac Enhancer Enriched” (“CEE”) motif. In three tested enhancers, the CEE motifs were necessary for their normal cardiac activity. Recently, independent experiments with one of these enhancers (associated with *mid*) have confirmed the supposition that the CEE motif may correspond to functional binding sites of ETS domain transcription factors (Benjamin Schwarz and Ingolf Reim, personal communication) [79].

Ahmad *et al.* (2014) [24] have extended this type of machine learning approach to predict cell type-specific cardiac enhancers in the *Drosophila* genome. For the classification, they used training sets of known enhancers that are specific for either the cardiac mesoderm (CM), myocardial cells (CCs), or pericardial cells (PCs), and additionally their orthologous sequences in other *Drosophila* and arthropod species. The binding motifs for the regulators Twist (in the early cardiac mesoderm), Tin (in the cardiac mesoderm and developing heart) and Mef2 (in myocardial cells) and the available ChIP data for these [22] were used as additional inputs, with random genomic sequences of similar GC content and length used as controls. When these classifiers were applied to the whole genome, the top 100 (and to a lesser degree top 1000) genes near the putative enhancers within each group were significantly associated with expression in CM, CCs, and PCs, respectively. All 28 cardiac enhancers reported by Jin *et al.* [23] were identified with the “binding motif + ChIP” classifier and about half of these were found with the “binding motif” classifier alone. Among 80 new candidates tested *in vivo*, about 1/3 were active in the cardiac mesoderm and/or heart cells, but because these were selected from a wide range of priority scores, it is not clear whether this success rate is higher than with a selection by ChIP data alone or by ChIP occupancy + Tin motifs, as described above. The predicted cardiac cell type specificity (or lack thereof) was observed with a portion of the tested enhancers, particularly those with a high priority score, thus showing some success in achieving the original goals. With regard to motif enrichment, it was found that, apart from the Twi, Tin, and Mef2 motifs used as classifiers, binding motifs for the GATA, zinc finger, LIM homeodomain, STAT families of transcription factors, as well as a motif potentially associated with the transcription factor Myb were enriched in enhancers that are active in all cardiac cell types. Furthermore, motifs for Su(H), a downstream effector of Notch signals, were enriched in PC-specific enhancers as opposed to CC-specific enhancers, thus providing hints to the possible involvement of both known and yet unknown factors during general or cell type-specific events of cardiogenesis. These hints were strengthened in one case by showing that the Myb motifs and *Myb* gene activity were required for full enhancer activity, and in another by documenting that the Su(H) motifs were required for suppressing enhancer activity in the CCs and thus restricting it to the PCs. Hence, in the latter case, Su(H) would only be required for Notch-independent repressive effects within the CCs, but not for promoting activation within the PCs. It remains to be shown whether this explanation is applicable more generally and whether there is Notch signaling between CCs (which display expression of the ligand Delta [80]) and PCs of the developing heart.

In a follow-up study, Busser *et al.* (2015) [19] used an enlarged training set of 73 enhancers that were curated from the literature and validated with regard to their expression in the CM, CCs, and PCs. As a reasonable number of these were active in specific subsets of PCs (Eve-PCs, Tin-PCs, Svp-PCs, or Odd-PCs [1]) the authors sought to define discriminating features not only for the principal cell types of the heart but also for the different subtypes of PCs. The support vector machine for classification was constructed by again using the available ChIP data with cardiogenic factors [21], together with >1000 TF binding motifs from databases. In addition, the published distributions of histone modifications were used, which however were derived from the whole FACS-isolated mesoderm and not specifically from heart tissues [62]. Interestingly, in a gene ontology (GO) analysis the genes associated with the cell type-specific candidate enhancers from each class that scored highly with this support vector machine showed enrichments for features that made biological sense. Thus, genes associated with enhancers predicted to be active in all heart cells (pan-PC/pan-CC) tended to be linked with developmental, signaling and transcriptional functions and those associated with enhancers with predicted expression in all CCs (pan-CC) tended to be linked with myogenic functions, including cell adhesion and the actin cytoskeleton. Genes associated with pan-PC enhancers were associated with renal system development, which correlates with the known role of these cells in ultrafiltration of hemolymph; those associated with predicted Odd-PC enhancers were enriched for chemotaxis and locomotion functions, which may be connected to the developing hemocytes in the Odd^+^ lymph gland; and genes associated with enhancers with predicted activity in Eve-PCs and Svp-PCs were enriched for extracellular matrix (ECM) annotations. Production of ECM for the stable attachment of PCs and alary muscles is a known function of PCs (e.g., [81]). Among 47 predicted cardiac enhancers that were tested *in vivo*, 19 (40%) were active in cardiac tissues, including several that were active in the correct cardiac cell type or PC subtype. It was observed that a meaningful interrogation of the epigenetic signatures of individual genomic loci would require analyzing homogenous subpopulations of cells, which has not been achieved to date. To identify sequence motifs with possible cell type specific functions within the developing heart, the authors aimed to identify sequence features that were positively weighted within a particular cardiac cell subtype classification and were depleted or irrelevant for the other cardiac subtype models. Five candidate motifs with this behavior were selected for functional verification in one representative enhancer for each. A particular type of zinc finger binding motif with high positive weight in the “Odd-PC” classification was found to be essential for the activity of an enhancer of the gene *mib1* in Odd-PCs. ETS-domain binding motifs with strong weight in the “Eve-PC” classification were needed for the activity of an enhancer associated with *Doc1* in Eve-PCs, This indicates that ETS-domain factors are not only important within the myocardial cells (as discussed above, [23]), but also in Eve pericardial cells (although it should be cautioned that the *Doc* genes are actually down-regulated in Eve-PC progenitors [69]). Likewise, motifs possibly corresponding to binding sites of the basic region/leucine zipper TF Pdp1, which were positively weighted among the “Odd-PC + Eve-PC” classification, were required for the activity of an enhancer in Odd-PCs and its repression in Eve-PCs. Two other motifs, possibly associated with the Metal regulatory factor 1 (MTF1) and the zinc finger factor Hamlet, respectively, and highly weighted among the “pan-PC” and “pan-CC” classifications, were required for full activity of another representative enhancer in all heart cells. The success rate of these verification experiments has not been reported, and detailed follow-up experiments that include rigid genetic verifications are required for proving any roles of the suggested factors. Nevertheless, these results provide further encouragement that, particularly after further extensions and refinements, cell type-specific sequence features and corresponding trans-acting factors with functional importance can be identified through approaches of this type.

## 6. Conclusions

**Figure 2:**
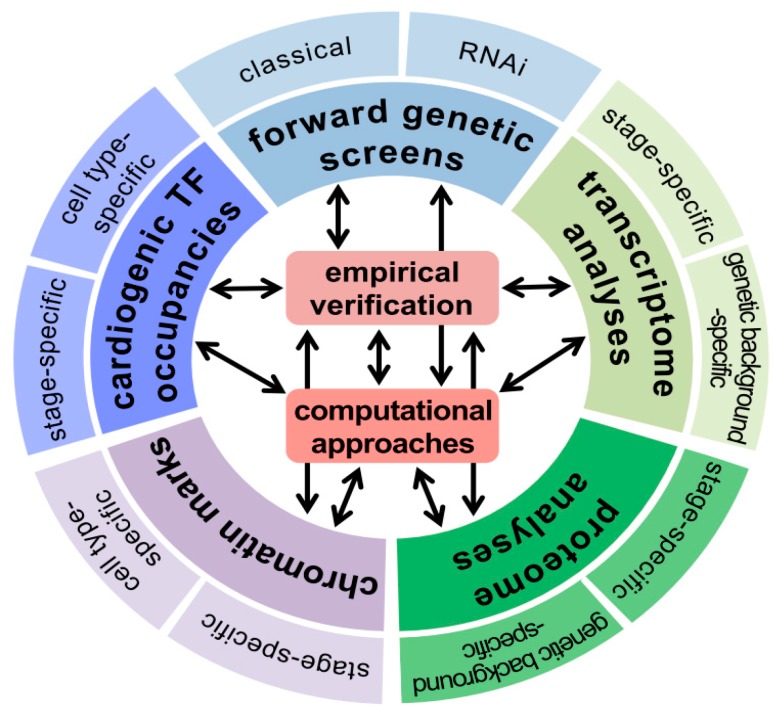
Integration of genome-wide experimental approaches with computational and empirical studies in *Drosophila* cardiogenesis research.

During recent years, global screens, which aimed to interrogate the entire *Drosophila* genome either by functional or by molecular criteria that are pertinent to heart development, have produced large troves of new data. These datasets, and follow-up studies propelled by them, have already shed new light on important steps as well as on global events of *Drosophila* heart development, and have thereby expanded our picture of this event significantly. Even greater amounts of invaluable information can still be extracted from these datasets by in-depth functional studies *in vivo* and *in vitro*, as well as by integrating and interrogating them with increasingly sophisticated computational approaches. Newly developed genetic tools employing CRISPR/Cas9 methodologies [82] and more stringently controlled RNAi libraries [5] will facilitate and expedite this work. Careful *in vivo* verification of the predictions made in initial rounds of machine learning is crucial for providing solid data inputs during successive rounds, which will increase the statistical power and make the computational predictions more and more accurate. These types of reiterative strategies, together with the continuing refinement of genomic methodologies including the possibility of interrogating homogeneous populations of *Drosophila* heart cells or single cells, are expected to greatly advance our understanding of *Drosophila* cardiogenesis during the coming years (Figure 2).

## Figures and Tables

**Table 1:** List of the genome-wide studies related to *Drosophila* heart development discussed in the text.

**Mutational Screens**	**Type**	**Heart Markers Used**	**Chromosome**
Tao *et al.*, 2007 [7]	*deficiencies*	*tup-GFP* or *Hand-GFP*	2nd
Yi *et al.*, 2006 [8]	*avail. lethals*	*Hand-GFP*	various
Hollfelder *et al.*, 2014 [9,10]	*EMS*	*tin-GFP + org-1-RFP*	2nd
Drechsler *et al.*, 2013 [11]	*EMS (Zuker)*	*Hand-GFP*	2nd, 3rd
**RNAi Screens**	**Type**	**Tissue**	
Kim *et al.*, 2004 [12]	injections	embryonic heart (*Mef2-lacZ*-marked)	
Neely *et al.*, 2010 [13]	GAL4-induced	developing adult heart	
**Transcriptome, Proteome Screens**	**Type**	**Tissue**	**Genotypes**
Zeitouni *et al.*, 2007 [14]; Potier *et al.*, 2014 [15]	transcriptome	developing adult heart	WT
Cammarato *et al.*, 2011 [16]	proteome	adult heart	WT
Hallier *et al.*, 2015 [17]	transcriptome	larval heart 3rd instar	*Hand*/WT
Ahmad *et al.*, 2012 [18]; Busser *et al.*, 2015 [19]	transcriptome	embryo, dorsal mesoderm	various heart mutants/WT
**Chromatin Immunoprecipitation Screens**	**Type**	**Transcription factors**	
Liu *et al.*, 2009 [20]	ChIP-chip	Tin	
Junion *et al.*, 2012 [21]; Zinzen *et al.*, 2009 [22]	ChIP-chip	Tin, Doc, Pnr, Mad, dTCF	
Jin *et al.*, 2013 [23]	ChIP-chip	Tin	
**Computational Screens**	**Type**	**Aims**	
Ahmad *et al.*, 2014 [24]; Busser *et al.*, 2015 [19]	machine learning	cardiac enhancer identification	

## Abbreviations

The following abbreviations are used in this manuscript:

CC	Cardial Cell (aka, myocardial cell, cardioblast)
ChIP	Chromatin Immunoprecipitation
CM	Cardiogenic Mesoderm
CRISPR/Cas9	Clustered Regularly Interspaced Short Palindromic Repeats
CRM	*Cis*-Regulatory Module
ECM	Extracellular Matrix
PC	Pericardial Cell
TF	Transcription Factor
